# Utilizing a Kidney-Targeting Peptide to Improve Renal Deposition of a Pro-Angiogenic Protein Biopolymer

**DOI:** 10.3390/pharmaceutics11100542

**Published:** 2019-10-18

**Authors:** Fakhri Mahdi, Alejandro R. Chade, Gene L. Bidwell

**Affiliations:** 1 Department of Neurology, University of Mississippi Medical Center, Jackson, MS 39216, USA; fmahdi@olemiss.edu; 2 Department of Physiology and Biophysics, University of Mississippi Medical Center, Jackson, MS 39216, USA; achade@umc.edu

**Keywords:** elastin-like polypeptide, kidney-targeting peptide, vascular endothelial growth factor, drug delivery, renal targeting

## Abstract

Elastin-like polypeptides (ELP) are versatile protein biopolymers used in drug delivery due to their modular nature, allowing fusion of therapeutics and targeting agents. We previously developed an ELP fusion with vascular endothelial growth factor (VEGF) and demonstrated its therapeutic efficacy in translational swine models of renovascular disease and chronic kidney disease. The goal of the current work was to refine renal targeting and reduce off-target tissue deposition of ELP–VEGF. The ELP–VEGF fusion protein was modified by adding a kidney-targeting peptide (KTP) to the N-terminus. All control proteins (ELP, KTP–ELP, ELP–VEGF, and KTP–ELP–VEGF) were also produced to thoroughly assess the effects of each domain on in vitro cell binding and activity and in vivo pharmacokinetics and biodistribution. KTP–ELP–VEGF was equipotent to ELP–VEGF and free VEGF in vitro in the stimulation of primary glomerular microvascular endothelial cell proliferation, tube formation, and extracellular matrix invasion. The contribution of each region of the KTP–ELP–VEGF protein to the cell binding specificity was assayed in primary human renal endothelial cells, tubular epithelial cells, and podocytes, demonstrating that the VEGF domain induced binding to endothelial cells and the KTP domain increased binding to all renal cell types. The pharmacokinetics and biodistribution of KTP–ELP–VEGF and all control proteins were determined in SKH-1 Elite hairless mice. The addition of KTP to ELP slowed its in vivo clearance and increased its renal deposition. Furthermore, addition of KTP redirected ELP–VEGF, which was found at high levels in the liver, to the kidney. Intrarenal histology showed similar distribution of all proteins, with high levels in blood vessels and tubules. The VEGF-containing proteins also accumulated in punctate foci in the glomeruli. These studies provide a thorough characterization of the effects of a kidney-targeting peptide and an active cytokine on the biodistribution of these novel biologics. Furthermore, they demonstrate that renal specificity of a proven therapeutic can be improved using a targeting peptide.

## 1. Introduction

Elastin-like polypeptides (ELP) are a class of protein biopolymers composed of repeating five-amino acid units (VPGxG, where x is any amino acid except proline) with unique physical properties [1] and many advantages as drug carriers [2]. ELPs are relatively biologically inert, having little to no cytotoxicity [3,4,5] and low immunogenicity [6,7]. Also, being proteins rather than chemically synthesized polymers, they degrade in vivo into non-toxic natural amino acids [8]. ELPs also have a unique physical property of being thermally responsive. ELPs are highly soluble in aqueous solution below a distinct transition temperature, and they form coacervates and precipitate above the transition temperature. This aggregation process is fully reversible, and the transition temperature at which it occurs can be precisely tuned by changing the hydrophobicity of the guest residue in the VPGxG repeat or by changing the number of repeats [9]. This tunable phase transition makes ELPs extremely versatile as drug delivery platforms via three major strategies: ELPs with transition temperatures below body temperature can be used as slow-release drug depots, ELPs with transition temperatures just above body temperature can be used for thermally targeted drug delivery, and ELPs with high transition temperatures (above body temperature) can be used as soluble protein carriers for therapeutics.

ELPs designed with transition temperatures below body temperature form coacervates at the injection site after delivery in vivo. This was utilized to achieve slow-release drug depots [10,11]. For example, an ELP fusion with multiple copies of glucagon-like peptide 1 with a transition temperature below body temperature achieved slow release of GLP-1 over the course of 10 to 17 days in mice and monkeys [12], and controlled plasma glucose levels for five to ten days in multiple mouse models [10,12,13] of diabetes. In another application, an ELP–lacritin fusion protein with a low transition temperature formed a depot after injection into the lacrimal gland and enhanced tear production in mice [14]. It is also possible to design di-block ELPs containing a hydrophobic, low-transition temperature block and a hydrophilic, high-transition temperature block, which form nanoparticles at physiologic temperatures [15]. These constructs were used for chemotherapy and anti-cancer peptide delivery [16,17], ocular drug delivery [18], and immunotherapy [19], among others. If the transition temperature is tuned just above body temperature, ELPs can be used as carriers for thermally targeted drug delivery. In these applications, ELP-fused drugs were administered systemically and circulate as soluble proteins. However, at a target site (most development in this area was regarding tumor targeting) where external mild hyperthermia was applied, the ELPs formed coacervates and accumulated, resulting in enhanced delivery [20,21,22,23,24]. Finally, ELPs with transition temperatures well above body temperature can be used as soluble carriers for many types of therapeutic agents. ELP fusion to small peptides, proteins, or small molecule drugs can increase solubility [25,26], protect the cargo from degradation [27,28], slow plasma clearance [29], and alter biodistribution [29], overall resulting in improved bioavailability. In addition to drug or therapeutic attachment, ELPs can be modified with targeting agents [4,30,31] or cell-penetrating peptides [5] to alter biodistribution [4] and intracellular distribution [32]. The pharmacological properties of soluble ELP-based biologics are also tunable. The plasma half-life of soluble ELPs is directly proportional to their molecular weight [33,34], allowing unique control over the in vivo clearance time.

Our lab focused on the development of in vivo soluble (high transition temperature) ELP fusion proteins as novel biologics. In one application, we tested an ELP fusion with vascular endothelial growth factor (VEGF), a pro-angiogenic cytokine [29,35,36]. VEGF is a potent inducer of angiogenesis and a stimulator of endothelial cell function [37], and reduced VEGF availability has been implicated in several disease states, including ischemic renal diseases [38,39] and preeclampsia [40]. VEGF supplementation therapy, however, is limited by the rapid plasma clearance of the small protein [41], the need for direct infusion into target tissues [42,43], and the ubiquitous nature of this cytokine that may lead to off-target effects. To overcome some of these limitations, we first developed an ELP fusion protein with human VEGF-A_121_ [29], the smallest, non-heparin-binding form of VEGF-A [44,45]. In extensive preclinical testing, the therapeutic potential of the ELP–VEGF fusion protein was demonstrated for treatment of kidney disease, including renal artery stenosis-induced renovascular disease [36,46,47] and chronic kidney disease [48]. These models, as observed in humans with these diseases, displayed a progressive loss of renal function associated with extensive microvascular rarefaction and renal fibrosis. ELP–VEGF was capable of targeting the kidney after either direct intra-renal injection [36] or systemic intravenous injection [46], improved microvascular density, induced increases in renal blood flow and the glomerular filtration rate, and reduced renal fibrosis. Furthermore, ELP–VEGF also improved renal outcomes in renovascular disease when used in combination with renal angioplasty and stenting [47].

Given the promise of ELP–VEGF for renal therapy, we are currently working to further optimize the therapeutic protein for renal disease treatment. Pasqualini et al. described a series of peptides that homed preferentially to the kidney or to the brain [49]. Using a phage display screen in mice, two peptides were identified that were enriched three- to five-fold in the kidney relative to the brain. The authors found that phages expressing one of these peptides were present at high levels in the glomerulus and between tubules [49], likely reflecting their target-binding in the renal vasculature. Previously, we showed that this kidney-targeting peptide (KTP), discovered by Pasqualini et al., increased the renal deposition of ELP when fused at ELP’s N-terminus about five-fold in both rats and pigs, while not affecting ELP levels in other organs [4]. The addition of KTP also slowed the plasma and whole-body clearance of ELP. Hence, we aimed to further improve the kidney-targeting of ELP–VEGF by incorporating the KTP technology. In this study, a kidney-targeted form of the ELP–VEGF protein was generated by fusing KTP to its N-terminus. All control proteins lacking each domain (ELP, KTP–ELP, and ELP–VEGF), plus non-ELP-fused VEGF, were generated in order to determine the effects of each domain on the in vitro activity and in vivo pharmacology of the molecule. After purifying all proteins, primary human glomerular microvascular endothelial cells were used to assess the in vitro potency of each protein, and the in vivo pharmacology of each protein (pharmacokinetics, biodistribution, kidney concentrations, and intrarenal distribution) was assessed in a mouse model.

## 2. Materials and Methods

### 2.1. Protein Expression and Purification

The ELP domain used in this study was ELP–[V_1_G_7_A_8_]-160, an ELP containing 160 VPGxG repeats, where x is Val, Gly, or Ala in a 1:7:8 ratio [20] (previously referred to as ELP2 [20,22,50], and referred to throughout this manuscript simply as ELP). This ELP has a molecular weight of approximately 61 kDa and a transition temperature of approximately 60 °C, making it a mid-sized ELP that does not aggregate in vivo, making it ideal as a soluble drug carrier. To generate ELP fusion proteins, the ELP coding sequence was modified by fusing an *E. coli* codon-optimized coding sequence for human VEGF–A_121_ in frame at the ELP C-terminus (as described in [29]) and/or fusing a coding sequence for a short kidney-targeting peptide [49] at the N-terminus (as described in [4]). The resulting constructs (ELP, KTP–ELP, ELP–VEGF, and KTP–ELP–VEGF) were expressed in *E. coli* and purified by inverse transition cycling, as previously described [29,50]. Free human VEGF–A_121_ was purchased from ProSpec (East Brunswick, NJ, USA).

### 2.2. Determining the Transition Temperature of ELP Fusion Proteins

Each ELP fusion protein was dissolved in phosphate-buffered saline at a final concentration of 10 μM. Turbidity of the ELP protein solutions was measured by monitoring optical density at 350 nm (OD_350_) using a UV–visible spectrophotometer with a Peltier-controlled temperature block (Cary 100, Agilent, Santa Clara, CA, USA). The temperature was increased from 20 °C to 90 °C at a rate of 0.5 °C per minute and data were collected every 0.5 °C with an average time of 2 s. Turbidity data were plotted as the percentage of the maximum OD_350_ after correcting the baseline to zero at 20 °C. A plot of the first derivative of the turbidity profile was generated using Graphpad Prism (GraphPad Software, Inc., San Diego, CA, USA). The transition temperature (T_t_) was defined as the peak in the first derivative plot of the aggregation curve.

### 2.3. Cell Culture

Human glomerular microvascular endothelial (HGME) cells were purchased from Cell Systems (Kirkland, WA, USA) and subcultured according to the manufacturer’s recommendations using Attachment Factor^TM^ (Cell Systems, Kirkland, WA, USA) and complete classic medium supplemented with Culture Boost^TM^ (Cell Systems, Kirkland, WA, USA). Cells in passage 4–13 were used for all experiments. Human renal proximal tubular epithelial cells (HRPTEpC) were purchased from Cell Applications, Inc. (San Diego, CA, USA) and subcultured according to the manufacturer’s recommendations using RenaEpi Growth factor media. Cells in passage 2–4 were used for all experiments. Human podocyte cells were purchased from Celprogen (Torrance, CA, USA) and subcultured according to the manufacturer’s recommendations using human podocyte cell culture media plus serum. The cells were seeded in ECM-coated flasks or Microtiter plates purchased from Celprogen. Cells in passage 9–13 were used for all experiments. All cells were maintained at 37 °C in a humidified incubator at 5% CO_2_.

### 2.4. Labeling Polypeptides with Fluorescent Probes

ELP and KTP–ELP were labeled on an engineered cysteine residue either with fluorescein-5-maleimide (for flow cytometry experiments) or tetramethyrhodamine-5-maleimide (for in vivo experiments), as previously described [50]. ELP–VEGF and KTP–ELP–VEGF were labeled on primary amine residues (which did not interfere with the ability of VEGF to bind its receptor) using 5-FAM (5-carboxyfluorescein, succinimidyl Ester) or NHS-Rhodamine (5/6-carboxy-tetramethyl-rhodamine succinimidyl ester) (Molecular Probes), as previously described [29]. The labeling efficiency was determined spectrophotometrically, as described [50].

### 2.5. Western Blotting and Silver Staining

ELP, KTP–ELP, ELP–VEGF, and KTP–ELP–VEGF proteins were electrophoresed using SDS-PAGE (4%–20%) Stain-Free^TM^ gels (Bio-Rad, Hercules, CA, USA). After electrophoresis, one of two identical gels was imaged by Bio-Rad stain-free imaging or processed for silver staining (Pierce), and the second gel was used for Western blotting. The proteins were transferred to a nitrocellulose membrane using 1-Step transfer buffer (Thermo Scientific, Rockford, IL, USA) with a Pierce G2 Fast Blotter (Thermo Scientific, Rockford, IL, USA). Following the transfer, the membrane was blocked with 5% dry milk in PBS-T for 1 h at room temperature. At the end of the incubation, the membrane was probed with anti-VEGF (A20) antibody (Santa Cruz, SC 152) at 1:200 dilution overnight at 4° C. Following incubation, the membrane was washed with PBS-T. After the wash, the membrane was incubated with goat anti-rabbit poly-HRP antibody (Pierce) at a 1:10,000 dilution for 1 h at room temperature. Following the incubation, the membrane was washed with PBS-T, incubated with SuperSignal West Femto substrate (ThermoFisher, Waltham, MA, USA), and the bands were visualized using chemiluminescence. Blot imaging was performed using the Bio-Rad Universal Hood Gel Doc System.

In another set of experiments, cell lysates of HGME, HRPTEpC, and human podocytes were prepared using radioimmunoprecipitation assay (RIPA) buffer. Cell lysates at equal concentrations were electrophoresed on SDS-Page (4%–20%) Stain-Free^TM^ gels (Bio-Rad). After electrophoresis, the proteins were transferred to a nitrocellulose membrane. The membranes were blocked with 5% dry milk in PBS-T for 1 h at room temperature. The membranes were probed for anti-VEGF R1 (Abcam, ab 23152, Cambridge, MA, USA) and anti-VEGF R2 (D8, Santa Cruz Biotechnology, Santa Cruz, CA, USA) at 1:1000 and 1:200 dilutions, respectively. The membranes were incubated with primary antibodies overnight at 4 °C. At the end of incubation, the membranes were washed with PBS-T, followed by incubation with anti-rabbit poly-HRP at 1/10,000 dilution for 1 h at room temperature. Blots were visualized as described above, then re-probed with anti-GAPDH antibody (Millipore, MAB374).

### 2.6. Proliferation Assay

HGME cells were seeded at 10,000 cells/well in 96-well plates and incubated at 37 °C in a humidified incubator with 5% CO_2_ overnight. The cells were serum and growth factor starved for 2–3 h before treatment. After starvation, 100 μL of each protein (ELP, KTP–ELP, ELP–VEGF, KTP–ELP–VEGF, and free VEGF) was added to basal media to make final concentrations of 1, 10, and 100 nM, and incubated for an additional 72 h. Viable cells were detected using MTS cell proliferation assay (Promega). The data shown represent the mean ± standard error of the mean (s.e.m.) of three independent experiments each performed in quadruplicate.

### 2.7. Tube Formation Assay

A 48-well plate, which was sterile and non-tissue culture treated, was coated with growth factor-reduced Matrigel (BD Biosciences). HGME cells were serum- and growth factor-starved for 2–3 h before seeding them over Matrigel-coated wells at 30,000 cells per well in 5% complete media containing 0.1 mg/mL of heparin in the absence or presence of a final concentration of 100 nM of the proteins ELP, KTP–ELP, ELP–VEGF, KTP–ELP–VEGF, or free VEGF. The cells were incubated at 37 °C in a humidified incubator with 5% CO_2_ for 5 h. At the end of the incubation, the cells were imaged with an inverted microscope using bright field illumination and 10× magnification. Five non-overlapping fields per well were imaged, and the tubes between two cell nodes were counted for each field, averaged for each well, and expressed relative to untreated wells. The data represent the mean ± s.e.m. of three independent experiments.

### 2.8. Migration Assay

Corning BioCoat growth factor-reduced Matrigel Invasion Chambers (Corning Biocoat) were warmed to room temperature, and the interior of the inserts were rehydrated with basal media (Cell Systems) for 2 h in a humidified incubator at 37 °C with 5% CO_2_. HGME cells at 30,000 cells per well in basal media containing 1% fetal bovine serum and 0.1 mg/mL heparin were added to the interior of the inserts in 500 μL volume. ELP, KTP–ELP, ELP–VEGF, KTP–ELP–VEGF, and free VEGF at a final concentration of 100 nM in a final volume of 750 μL was added to the same media in the wells of a 48-well tissue culture-treated plate. The inserts were gently placed into each designated well, taking care to avoid air bubbles. The cells were incubated for 16–18 h in a humidified incubator at 37 °C with 5% CO_2_. After incubation, any cell suspension left in each insert was removed, the inserts were rinsed with DPBS, and non-invading cells were scrubbed from the upper surface of the membrane using a cotton swab. The cells on the lower surface of the membrane were stained with 0.1% crystal violet in 10% ethanol at room temperature for 30 min. The inserts were rinsed with water and air dried for an additional 60 min. Membranes were photographed using an inverted microscope and 10× magnification objective on five independent fields per membrane. The number of cells per field were counted and averaged for each well. The data represent the mean ± s.e.m. of three independent experiments.

### 2.9. Flow Cytometry

HGME, HRPTEpC, and human podocyte cells were seeded at 300,000 cells per well in 6-well plates (ECM-coated plates were used for human podocyte cells) and incubated at 37 °C in a humidified incubator with 5% CO_2_ overnight. The cells were washed and treated with fluorescein-labeled ELP, KTP–ELP, ELP–VEGF, and KTP–ELP–VEGF at a final concentration of 10 μM and incubated at 37 °C in a humidified incubator with 5% CO_2_ overnight. At the end of the incubation, the cells were washed with DPBS twice and 500 μL of cell-stripper buffer (Corning Mediatech, Tewksbury, MA, USA) was added to each well, followed by the addition of 1 mL of DPBS. The cell suspension was removed, placed in fresh polystyrene tubes, and centrifuged at 400× *g*. The cell pellets were resuspended in 400 μL of DPBS. The relative green fluorescence intensity of the cells was measured using flow cytometry (Gallios, Beckman Coulter, Indianapolis, IN, USA). Forward versus side scatter was used to gate viable cells, and the mean fluorescence intensity was determined. The mean fluorescence intensity was corrected for autofluorescence (determined by analyzing untreated cells) and normalized by correcting for differences in labeling efficiency among the various proteins. Independent experiments were performed in duplicate and repeated thrice.

### 2.10. In Vivo Biodistribution Studies

All animal studies were approved by the Institutional Animal Care and Use Committee of the University of Mississippi Medical Center (Approval number: 1379B, 25 April 2019), and the experiments were performed according to the Guide for the Care and Use of Laboratory Animals [51]. For acute tissue biodistribution studies, SKH-1 Elite hairless mice (female, Charles River) were anesthetized with 3% isoflurane, followed by administration of rhodamine-labeled ELP, KTP–ELP, ELP–VEGF, and KTP–ELP–VEGF (20 mg/kg) by intravenous injection (IV) into the femoral vein. Four hours after the injections, the mice were euthanized while still under anesthesia, and their organs were collected for whole-organ fluorescence biodistribution analysis (*n* = 4 mice per protein). All organs were imaged using an in vivo imaging system (IVIS Spectrum, Perkin Elmer, Foster City, CA, USA), followed by embedding in freezing medium (Tissue-Plus optimal cutting temperature (O.C.T.), Fisher scientific, Waltham, MA, USA) and flash freezing in dry ice/isopentane for further analysis.

For longer-term pharmacokinetic and whole-body fluorescence experiments, SKH-1 Elite hairless mice (*n* = 4 mice per protein) were injected with rhodamine-labeled ELP, KTP–ELP, ELP–VEGF, and KTP–ELP–VEGF intravenously (20 mg/kg, femoral vein), as above, and blood was sampled intermittently after injection by nicking the tail vein. Whole-animal fluorescence images of the live animals were collected at regular intervals for 120 h using an IVIS Spectrum (Perkin Elmer).

IVIS images were collected using 535 nm excitation and 580 nm emission filters, auto exposure, and small binning. Regions of interest (ROIs) were drawn over the entire organ (tissue biodistribution studies) or animal (whole-body clearance studies), and the mean radiant efficiency was determined. Standard curves of each protein were pipetted into a black 96-well plate, which were subsequently imaged with identical imaging parameters. The mean tissue fluorescence was fit to these standard curves to correct for any differences in labeling levels among the polypeptides.

Plasma samples from each blood collection were prepared by centrifugation, and 2 μL of each plasma sample was used to measure the fluorescence intensities for each labeled protein using a fluorescence plate reader and a NanoQuant plate (Tecan, Männedorf, Switzerland) with an excitation wavelength of 535 nm and an emission wavelength of 585 nm. The relative fluorescence measurements of the plasma samples were compared to a standard curve for each protein with known concentrations to determine the exact plasma concentrations of each protein at each time point. Plasma clearance data were fit to a two-compartment pharmacokinetic model, as described in [22].

Frozen kidneys from the acute biodistribution cohort described above were sectioned into 16 μm mid-hilar sections using a cryomicrotome. Standards of known quantities of each labeled protein were also frozen and sectioned to the same thickness, as described in [52]. For the quantitative histology assay, kidney sections and standards were scanned with a fluorescence slide scanner as described [52,53], and the mean fluorescence intensities of the sections were fit to the standard curves to determine the intra-renal concentrations of each protein. Next, the sections were fixed and processed for fluorescence histology by co-staining for CD31, an endothelial cell marker, or synaptopodin, a podocyte marker, and imaged by confocal microscopy, as described in [4].

### 2.11. Statistical Analysis

In vitro experiments were plated in replicates, as indicated above, and repeated at least three times independently. Data were analyzed using one-way ANOVA with post-hoc Tukey’s multiple comparison to detect differences among proteins or two-way ANOVA with post-hoc Tukey’s multiple comparison to detect differences among protein treatment and dose, as appropriate, using Graphpad Prism. Biodistribution data were analyzed using two-way ANOVA with factors for protein treatment and organs, and a post-hoc Tukey’s multiple comparison correction was used.

## 3. Results

### 3.1. Production of ELP Fusion Proteins

**Figure 1:**
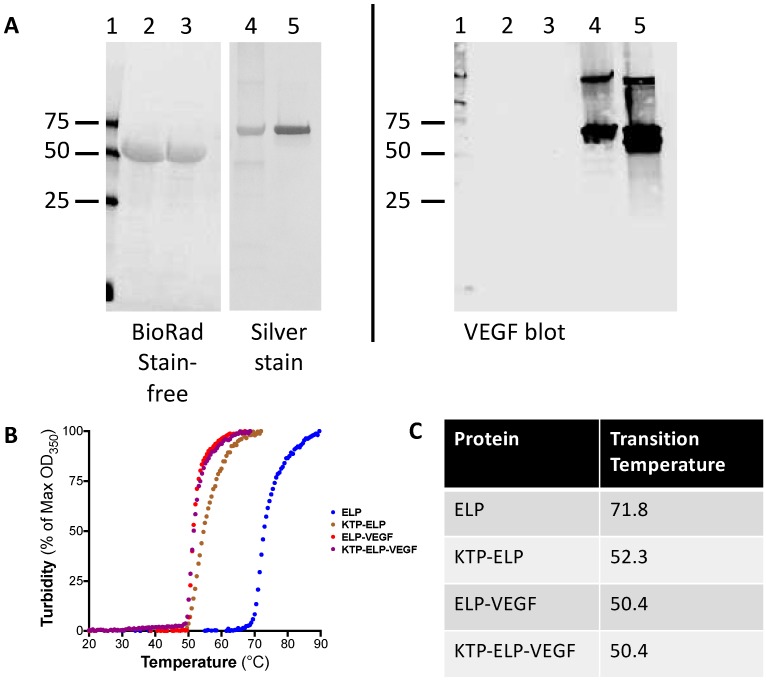
Purification of elastin-like polypeptide (ELP) constructs. (**A**) Proteins were purified using inverse transition cycling, and purity was assessed using stain-free imaging and silver staining (left panel). Confirmation of the vascular endothelial growth factor (VEGF) moiety in the ELP–VEGF and kidney-targeting peptide (KTP)–ELP–VEGF constructs was determined by Western blot (right panel). Lanes: 1—Marker, 2—ELP, 3—KTP–ELP, 4—ELP–VEGF, and 5—KTP–ELP–VEGF. (**B**) The transition temperature was determined by monitoring the turbidity of the solutions of each protein with increasing temperature. (**C**) The transition temperature was defined as the peak in the first derivative of the turbidity plot.

ELP, KTP–ELP, ELP–VEGF, and KTP–ELP–VEGF proteins were purified using inverse transition cycling. All of the proteins were obtained at high purity, as assessed by SDS-PAGE and stain-free or silver staining (Figure 1A, left panel). Yields of the proteins from the bacterial expression system varied according to the construct. ELP and ELP–VEGF were readily purified in mg to gram quantities, with yields >10 mg of protein per liter of bacterial culture. In contrast, KTP–ELP and KTP–ELP–VEGF were expressed by bacteria at much lower levels, typically less than 1 mg of protein per liter of bacterial culture. Stain-free imaging was used to visualize ELP and KTP–ELP proteins (lanes 1 and 2), as they did not stain with Coomassie and stained very poorly with silver, but were highly visible using Bio-Rad Stain-Free^TM^ technology. Silver staining worked well on the VEGF-modified proteins (lanes 3 and 4). Gel imaging revealed that all of the proteins were highly pure and electrophoresed at the expected molecular weights. Western blotting was used to confirm the presence of the VEGF moiety. A duplicate gel was probed for VEGF, and the ELP–VEGF and KTP–ELP–VEGF lanes produced strongly reactive bands that matched well to the silver-stained bands, with no VEGF reactivity in the ELP or KTP–ELP lanes. A higher molecular weight VEGF-reactive band was also visible in the ELP–VEGF and KTP–ELP–VEGF samples, and its molecular weight indicated that it represented a disulfide linked covalent dimer of ELP–VEGF or KTP–ELP–VEGF (due to the lack of complete reduction prior to electrophoresis). However, these dimer bands were a very minor component of the total protein, as assessed by silver staining. There also appeared to be a VEGF-reactive band at a molecular weight just below the full length KTP–ELP–VEGF band in the Western blot. The identity of this band was unknown, but it was not visible in the silver staining, suggesting that it was a very minor component of the total protein. These data illustrated the integrity and purity of the proteins and confirmed the presence of the VEGF moiety in the ELP–VEGF and KTP–ELP–VEGF proteins.

The transition temperature of all proteins was determined by monitoring the turbidity of the solutions with increasing temperature. As shown in Figure 1B, all ELP-containing proteins underwent a temperature-induced phase transition, resulting in production of polypeptide coacervates. The T_t_ of each protein was defined as the peak of a first-derivative plot of the turbidity curve. The unmodified ELP had a transition temperature of 71.8 °C (Figure 1C). Modifications of the ELP carrier with the KTP or VEGF moieties caused large decreases in the T_t_. The T_t_ of KTP–ELP was 52.3 °C, and the T_t_ of both ELP–VEGF and KTP–ELP–VEGF was 50.4 °C. Importantly, for all ELP fusion proteins used in this study, the T_t_ was well above physiologic temperature, suggesting that when injected in vivo, all of the ELP fusion proteins described here were present as soluble proteins and underwent coacervation.

### 3.2. ELP-Fused VEGF Constructs Stimulate Angiogenic-Like Activity in Human Glomerular Microvascular Endothelial Cells

**Figure 2:**
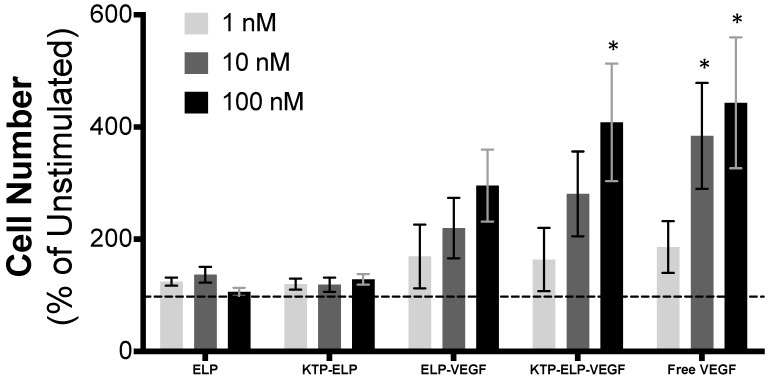
ELP-fused VEGF constructs stimulate endothelial cell proliferation. Human glomerular microvascular endothelial cells were exposed to basal media plus the indicated concentration of each protein for 72 h. The cell number was determined by MTS assay and expressed as a percentage increase relative to unstimulated cells (dashed line). * Statistically significant increase relative to unstimulated cells (two-way ANOVA with post-hoc Tukey’s multiple comparison).

In order to determine if the ELP–VEGF and KTP–ELP–VEGF fusion proteins maintained their VEGF signaling activity, their ability to stimulate proliferation, tube formation, and extracellular matrix invasion in HGME cells and their potency relative to free VEGF–A_121_ were assessed. VEGF is a potent mitogen and a chemokine for endothelial cells. When HGME cells were exposed to VEGF over the course of a 72 h experiment, proliferation was significantly stimulated in a dose-dependent manner (Figure 2). There were four-fold more viable cells after 72 h of exposure to VEGF versus unstimulated cells. Similar to free VEGF, ELP–VEGF and KTP–ELP–VEGF both induced HGME proliferation. The dose response was similar for all three proteins, as there were no statistically significant differences among the levels of stimulation induced by each protein within each dosage. In contrast, the ELP protein alone and the KTP–ELP protein that lacked the VEGF domain had no effect on HGME proliferation. These data were consistent with our previous results regarding ELP–VEGF in human umbilical vein endothelial cells [29] and HGME cells [36], clearly showing that fusion of VEGF to ELP or KTP–ELP carriers did not affect its potency to stimulate proliferation in endothelial cells.

**Figure 3:**
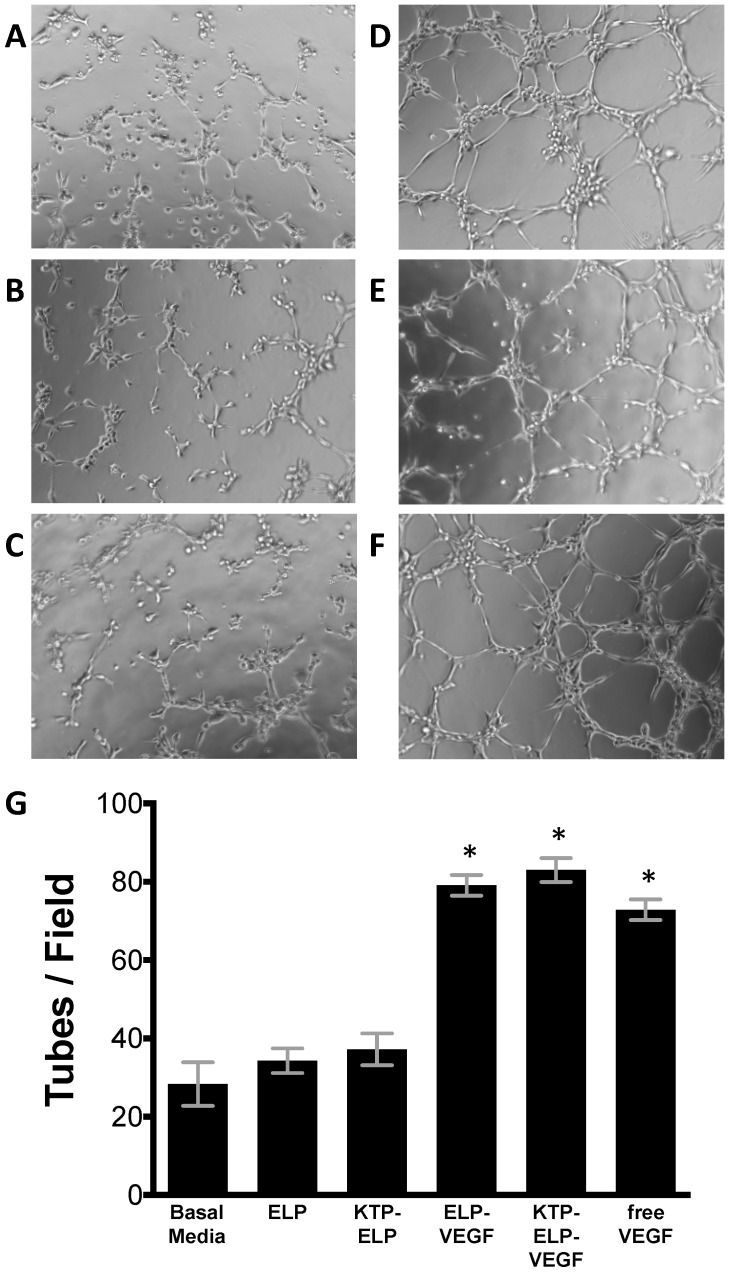
ELP-fused VEGF constructs stimulate endothelial cell tube formation. Human glomerular microvascular endothelial cells were plated on growth factor-reduced Matrigel and exposed to basal media plus saline (**A**) or 100 nM ELP (**B**), KTP–ELP (**C**), ELP–VEGF (**D**), KTP–ELP–VEGF (**E**), or free VEGF (**F**) for 5 h. (**G**)**.** The number of tubes per field were averaged over six identical fields for each treatment group, and the experiment was repeated four times. * Statistically significant increase relative to unstimulated cells (one-way ANOVA with post-hoc Tukey’s multiple comparison).

In addition to mitogenic activity, pro-angiogenic activity of the proteins was determined by assessing their ability to induce HGME tube formation on growth factor-reduced Matrigel. Without protein treatment, HGME cells poorly formed tube-like structures on the growth factor-reduced matrix (Figure 3A). ELP and KTP–ELP had no effect on the number of tube like structures (Figure 3B,C). ELP–VEGF, KTP–ELP–VEGF, and free VEGF, on the other hand, strongly induced tube formation (Figure 3D–F). The average number of tubes per field was not different between the cells treated with ELP–VEGF, KTP–ELP–VEGF or free VEGF at an equimolar dose (Figure 3G).

**Figure 4:**
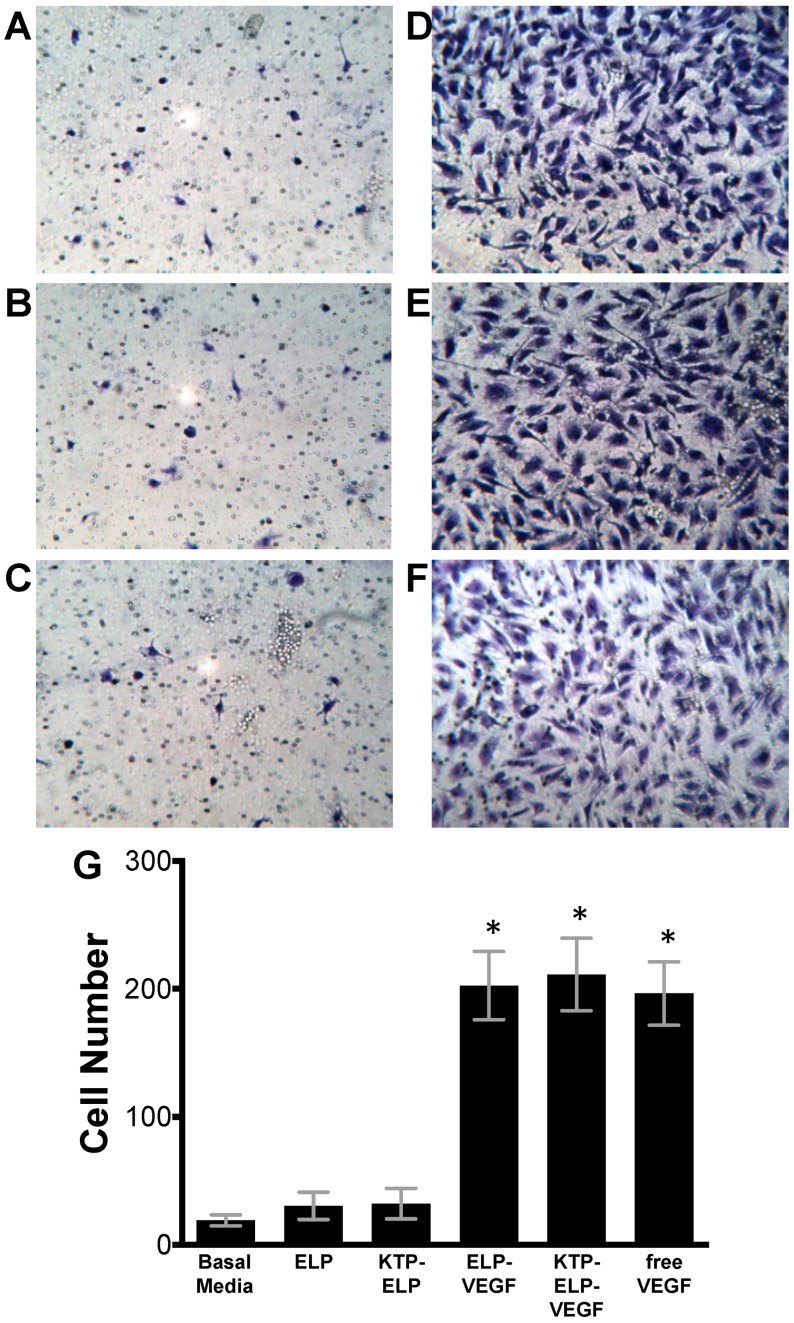
ELP-fused VEGF constructs stimulate endothelial migration. Human glomerular microvascular endothelial cells were plated in the top chamber of Matrigel-coated Boyden chambers in basal media and basal media plus saline (**A**) or 100 nM ELP (**B**), KTP–ELP (**C**), ELP–VEGF (**D**), KTP–ELP–VEGF (**E**), or free VEGF (**F**) was placed in the lower chamber. (**G**). Sixteen to eighteen hours after plating, the lower surface of the membrane was stained with crystal violet, and the number of cells per field were averaged over six identical fields for each treatment group. The experiment was repeated three times. * Statistically significant increase relative to unstimulated cells (one-way ANOVA with post-hoc Tukey’s multiple comparison).

The chemokine activity of the proteins was tested using HGME cells in a Boyden chamber Matrigel invasion assay. As shown in Figure 4, ELP–VEGF, KTP–ELP–VEGF, and free VEGF all induced significant invasion of HGME cells through the matrix toward the protein-containing chamber. No difference in the number of cells was seen between the three VEGF-containing protein groups. In contrast, ELP and KTP–ELP induced no endothelial cell Matrigel invasion.

### 3.3. Cell Binding/Uptake of Polypeptides in Primary Human Renal Cells

**Figure 5:**
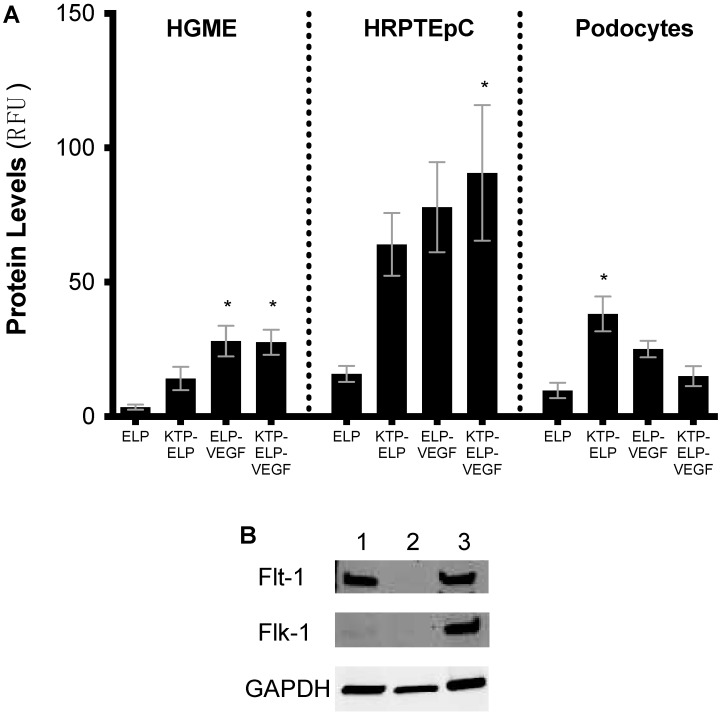
In vitro cellular binding/uptake of all constructs in primary human renal cells. Primary human glomerular microvascular endothelial cells (HGME), proximal tubule epithelial cells (HRPTEpC), and podocytes were exposed to each fluorescently labeled ELP fusion protein at 10 µM for 24 h. Protein levels bound to or in each cell type were determined by flow cytometry (**A**). Expression of the VEGF receptors Flt-1 and Flk-1 were determined in each cell line by Western blot ((**B**). Lane 1—HGME, Lane 2—HRPTEpC, and Lane 3—podocytes). * Statistically significant increase relative to ELP-treated cells (one-way ANOVA performed within each cell type with post-hoc Tukey’s multiple comparison).

After demonstrating that the ELP-fused VEGF fusion proteins maintained the ability to stimulate endothelial cells, primary human tubular epithelial cells and podocytes, as well as endothelial cells, were used to determine the contribution of each portion of the protein to cell-type binding. The addition of KTP to ELP increased its binding to all three renal cell types (Figure 5A), which was consistent with our previous results [4]. The proteins that contained the VEGF moiety, ELP–VEGF and KTP–ELP–VEGF, showed the most binding to endothelial cells. This was consistent with the VEGF moiety-mediating interaction with VEGF receptors, which were expressed by the endothelial cells (Figure 5B). Interestingly, KTP–ELP, ELP–VEGF, and KTP–ELP–VEGF all bound strongly to or were internalized by tubular epithelial cells, which did not express VEGF receptors (Figure 5A,B). This binding may have been due to interaction with other membrane proteins, including receptors responsible for protein reabsorption, which are a subject of ongoing studies. Binding to podocytes was increased by the addition of KTP to the ELP protein in the case of KTP–ELP, but not in the case of KTP–ELP–VEGF. The VEGF-containing proteins did not bind to podoctyes at levels higher than the ELP control, despite these cells expressing high levels of VEGF receptors. The reason for the lack of binding of the VEGF-containing proteins to podocytes was unclear, but it was consistent with the intra-renal distribution of these proteins (shown below).

### 3.4. Pharmacokinetics and Biodistribution of ELP, KTP–ELP, ELP–VEGF, and KTP–ELP–VEGF

**Figure 6:**
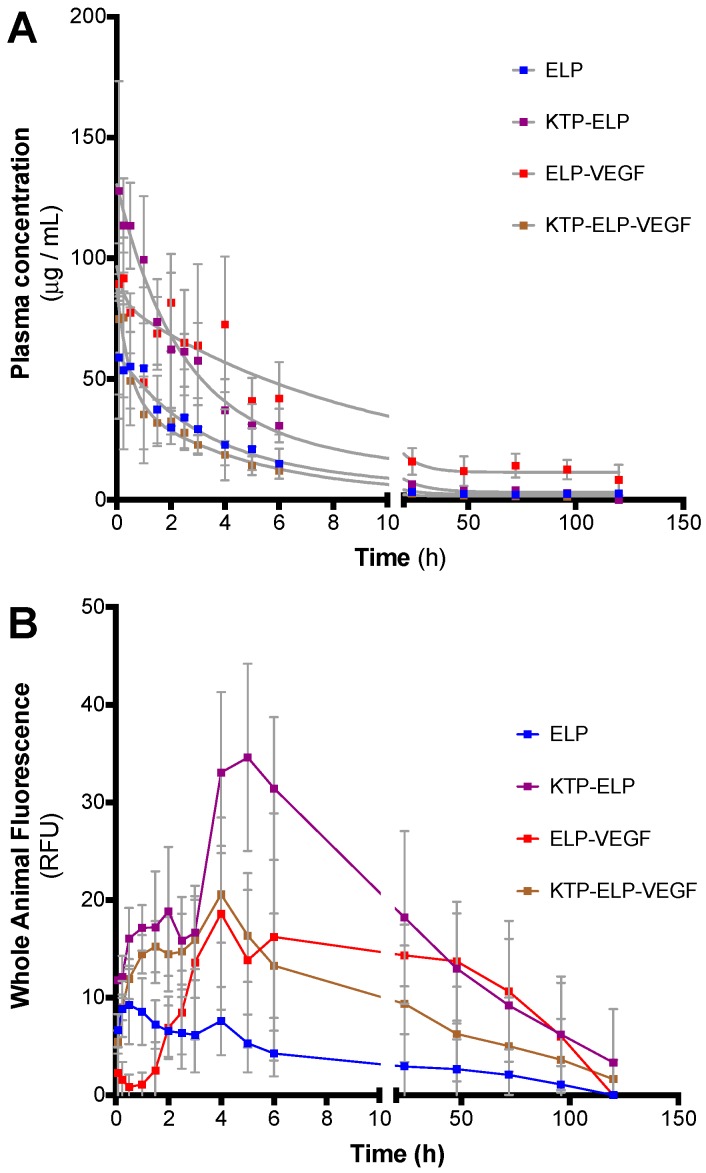
Pharmacokinetics of ELP fusion proteins. SKH-1 Elite mice were administered fluorescently labeled proteins via intravenous injection. Plasma samples and whole-body in vivo images were collected intermittently after injection to determine plasma clearance rate (**A**) and tissue clearance time (**B**).

The pharmacokinetics, whole-body clearance, and biodistribution of all proteins were determined in SKH-1 Elite hairless mice following a single bolus intravenous (IV) injection (20 mg/kg injected via the femoral vein). The plasma clearance kinetics were not significantly different among all polypeptides (Figure 6A). The terminal half-lives were 6.0, 8.0, 6.2, and 3.2 h for ELP, KTP–ELP, ELP–VEGF, and KTP–ELP–VEGF, respectively. Qualitative differences were seen in the whole-body clearance rates, which were determined by in vivo imaging of the hairless mice at each time point after polypeptide injection (Figure 6B). ELP peaked in the tissue most rapidly among the polypeptides tested, within 1 h of injection, then started to clear from the body. KTP–ELP peaked much later after injection (5 h) and remained at higher in vivo levels than ELP for approximately two days, which was consistent with our previous results in rats [4]. ELP–VEGF and KTP–ELP–VEGF also showed delayed peak tissue concentration times relative to ELP and were similar to each other with a peak at 4 h after injection. ELP–VEGF and KTP–ELP–VEGF cleared from the body with similar kinetics over the course of about three days. The slowed whole-body clearance of KTP–ELP, ELP–VEGF, and KTP–ELP–VEGF relative to the unmodified ELP was likely related to their binding to target molecules and/or extravasation in tissues. These data demonstrated that whole body clearance of KTP–ELP or ELP-modified VEGF proteins occurred over the course of two to three days, and they suggested that daily or every-other-day dosing would be ideal for future therapeutic applications if a repeated dosing regimen was required for a given disease indication.

**Figure 7:**
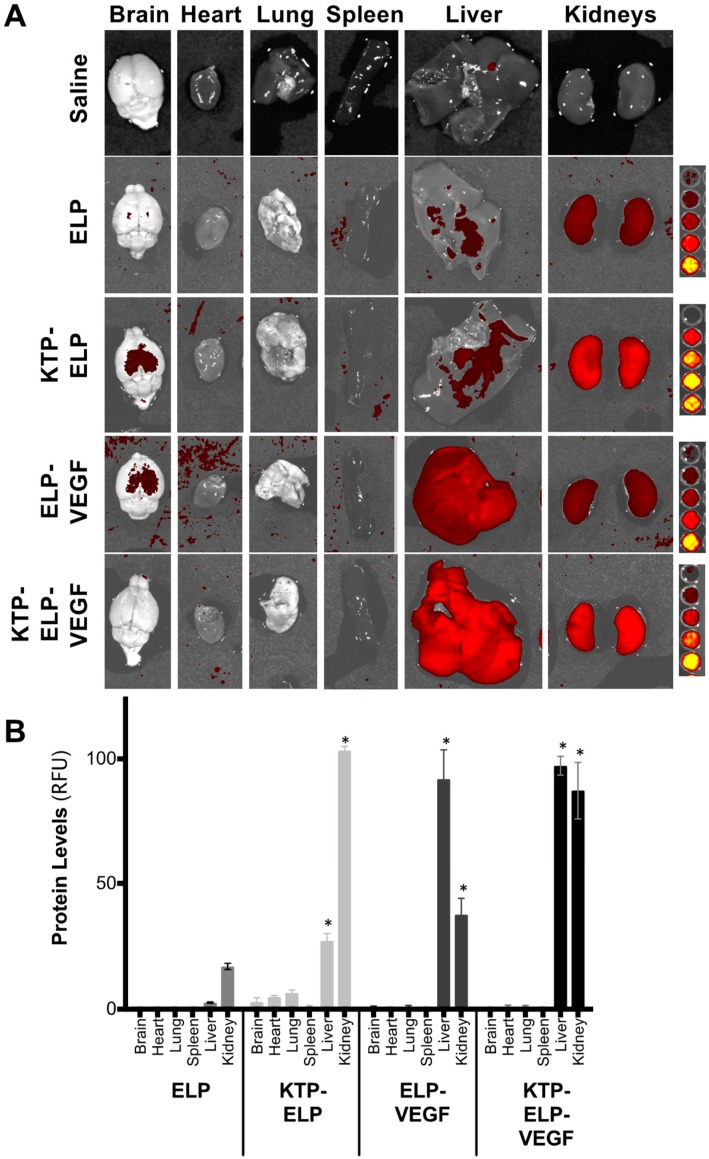
Biodistribution of ELP-fused VEGF constructs after intravenous (IV) administration in mice. SKH-1 Elite mice were administered rhodamine-labeled proteins via IV injection. Four hours after injection, their organs were harvested and analyzed using ex vivo fluorescence imaging. (**A**) Representative image of organs from each treatment group. (**B**) Organ levels were quantified by measuring the mean fluorescence intensity of each organ and fitting to a standard curve of the protein injectate. Data represent the mean ± s.e.m. of four mice per treatment. * Statistically significant difference relative to ELP (two-way ANOVA with post-hoc Tukey’s multiple comparison).

A separate cohort of mice was given an identical injection protocol, but these individuals were sacrificed 4 h after drug administration in order to determine organ biodistribution. Ex vivo whole-organ imaging (Figure 7A) revealed that, consistent with previous work in rats and pigs [4,23], ELP accumulated most in the kidney, and the addition of KTP to ELP increased renal deposition approximately five-fold (Figure 7B). The liver was a distant second in terms of organ levels of ELP and KTP–ELP, although KTP–ELP levels in the liver were significantly higher than ELP liver levels. ELP–VEGF demonstrated a different biodistribution profile. Its levels were highest in the liver, and it also accumulated at high levels in the kidney. The deposition of ELP–VEGF in the liver was consistent with our previous mouse study [29], although the biodistribution of ELP–VEGF appeared to be dependent on the species. In mice after a bolus IV injection, both in this study and in our previous work [29], ELP–VEGF accumulated at higher levels in the liver than in the kidney. However, in rats (after continuous intraperitoneal infusion [35], IV injection, or subcutaneous injection (unpublished data)) and in pigs after intravenous injection [46], ELP–VEGF accumulated in the kidneys at higher levels than in the liver. Interestingly, the addition of KTP to the ELP–VEGF construct redirected the protein toward the kidneys. Though the liver levels of KTP–ELP–VEGF remained high, KTP–ELP–VEGF was also present at equally high levels in the kidney. All polypeptide levels were low in the brain, heart, lung, and spleen, and there were no differences in these organs among the various proteins. These results demonstrated the ability of KTP to increase deposition of the ELP carrier in the kidney and to re-direct ELP–VEGF from the liver to the kidney. Future work will evaluate the effect of adding KTP to ELP–VEGF in other species where ELP–VEGF does not accumulate to such high levels in the liver.

**Figure 8:**
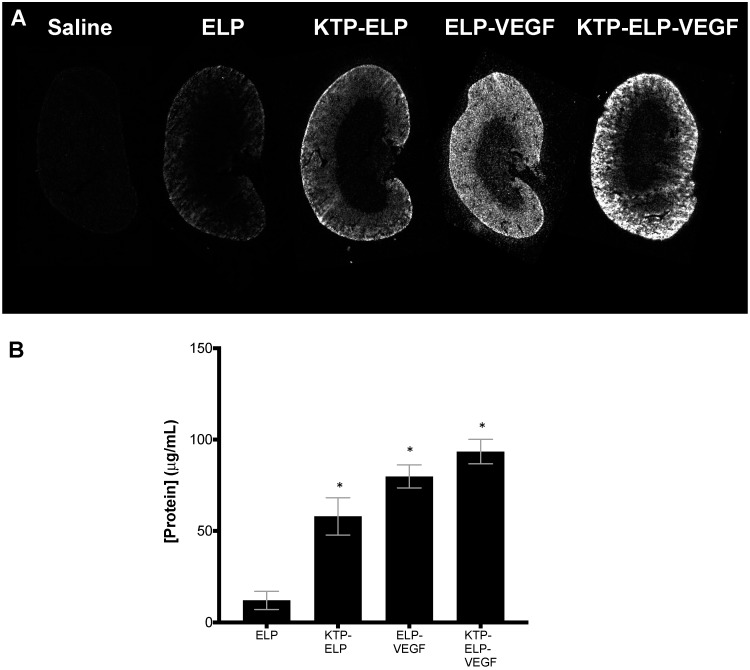
Renal levels of constructs after IV administration in mice as determined by quantitative fluorescence histology. SKH-1 Elite mice were administered rhodamine-labeled proteins via IV injection. (**A**) Four hours after injection, organs were harvested and cryosectioned, and sections were scanned with a fluorescent slide scanner. (**B**) Quantitative organ levels were determined by fitting the fluorescence intensity of kidney sections to standard curves cut to the same thickness as the tissues and scanned with identical parameters. Data represent the mean ± s.e.m. of four mice per treatment. * Statistically significant difference relative to ELP (one-way ANOVA with post-hoc Tukey’s multiple comparison).

In order to obtain more quantitative measures of intra-renal polypeptide concentrations and to determine intra-renal polypeptide distribution, the kidneys were cryosectioned and analyzed using direct fluorescence detection of the labeled polypeptides. Slice imaging of mid-hilar sections revealed that all polypeptides accumulated predominantly in the renal cortex (Figure 8A). Using a quantitative fluorescence histology assay [52], intra-renal concentrations were determined (Figure 8B). ELP levels were the lowest among the four proteins, with levels reaching approximately 12 μg/mL at this time point with this dosage. KTP–ELP, ELP–VEGF, and KTP–ELP–VEGF levels were all higher than ELP levels, ranging from 58–93 μg/mL at this dose and time point. In contrast to the whole-organ imaging, this more accurate assay did not reveal ELP–VEGF kidney levels to be lower than KTP–ELP or KTP–ELP–VEGF levels.

**Figure 9:**
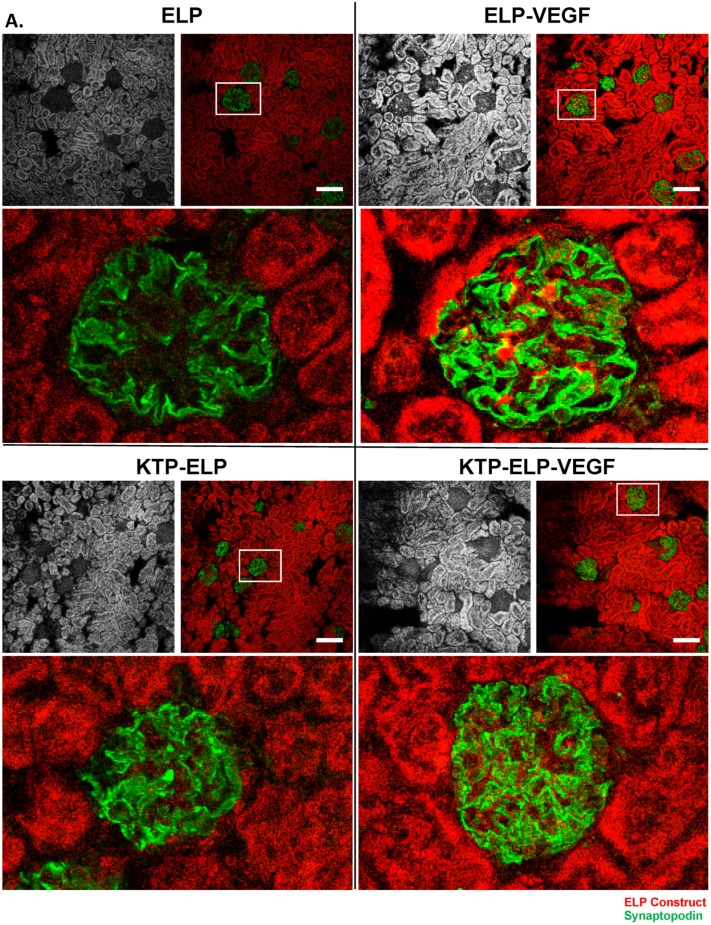

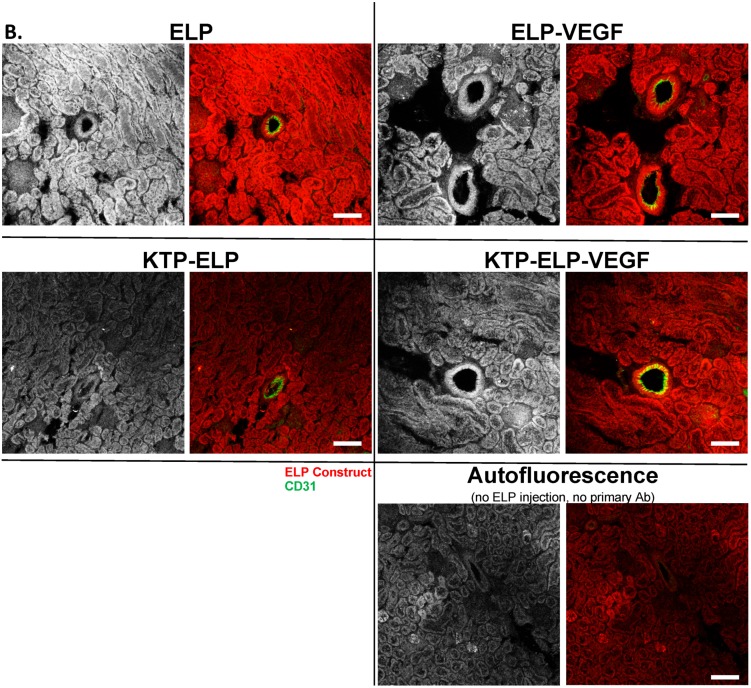
Intra-renal distribution of constructs. Localization of each polypeptide in the kidneys was determined by direct detection of its rhodamine label. The grayscale section shows ELP, KTP–ELP, ELP–VEGF, or KTP–ELP–VEGF localization in each section. Slices were co-stained with synaptopodin ((**A**), green) to mark podocytes or CD31 ((**B**), green) to mark endothelial cells. A saline-injected animal, with no primary antibody used in the staining, was used to determine autofluorescence levels in the red and green channels ((**B**), bottom panel). Scale bar = 100 μm.

Intra-renal histology was conducted with all proteins and overlaid with cell-type staining for podocytes (marked by synaptopodin staining, Figure 9A) or endothelial cells (marked by CD31 staining, Figure 9B). All of the polypeptides accumulated at high levels in the tubules. ELP and KTP–ELP were present at much lower levels in the glomeruli. However, ELP–VEGF and, to a lesser extent, KTP–ELP–VEGF were seen in punctate foci in the glomeruli (Figure 9A, insets). The focal glomerular staining of ELP–VEGF and KTP–ELP–VEGF did not directly overlay with synaptopodin staining, likely reflecting the presence of these proteins in the glomerular capillaries but not directly interacting with the podoctyes. When endothelial cells were co-stained, all of the proteins were visible in the walls of larger vessels (Figure 9B) in both the endothelial and vascular smooth muscle cell layers. Tubular staining of all proteins was also readily visible in these sections. To control for autofluorescence or non-specific antibody staining, sections from an animal injected with saline were stained using the same protocol, but with no primary antibody, and imaged with identical parameters. Autofluorescence was observed in the red channel at the settings used to detect the ELP proteins, especially in the tubules (Figure 9B, bottom panel). However, the autofluorescence did not reach the level of signal seen in the protein-treated animals, indicating that there were indeed ELP proteins present in the blood vessels and renal tubules. No staining occurred with the antibody control, thereby validating the specificity of the podocyte and endothelial cell markers. These data indicated that ELP proteins were present at high levels in the kidney in both the renal blood vessels and tubular epithelial cells. Additionally, the VEGF-containing proteins were present in focal regions within the glomeruli, consistent with the glomerular capillaries.

## 4. Discussion

The ELP molecule was shown to be a versatile drug carrier with many therapeutic advantages, including improved therapeutic targeting, controlled pharmacokinetics and/or drug release, and the ability to deliver many types of therapeutic cargo [2]. Our group has used ELPs extensively for the delivery of growth factors. We are currently developing ELP-fused growth factors, including members of the VEGF family, as therapeutics for kidney disease [54] and preeclampsia [55,56]. This work expanded on our previous studies, in which we demonstrated the ability of ELP fusion to facilitate VEGF purification [29], stabilize VEGF for in vivo delivery [29,36,46], and improve the efficacy of renal therapeutic angiogenesis [36,46,47]. In this work, we modified our previously-used ELP–VEGF fusion protein with a kidney-targeting peptide, and we determined the effects of each domain of the KTP–ELP–VEGF protein on its in vitro activity and cell binding and its in vivo pharmacokinetics and biodistribution.

In vitro studies in renal microvascular endothelial cells consistently showed that fusion of VEGF to the ELP carrier did not hamper its ability to stimulate angiogenic-like activities. Since the potency of endothelial cell proliferation, invasion, and tube formation were not affected by the fusion of VEGF to ELP carriers, we suspected that the chimeric proteins were able to engage with VEGF receptors at near-native affinity. Ongoing work will determine the affinity constants of the ELP-fused VEGF proteins for the Flt-1 and Flk-1 VEGF receptors, and compare the affinity to unmodified VEGF-A. Cell-binding studies also revealed interesting and somewhat unpredicted effects of each of the protein’s domains on cellular interaction. The VEGF-containing proteins were the highest binders to the endothelial cells, which was expected given the expression of VEGF receptors by these cells. Also consistent with expectations and previous studies was the ability of KTP to improve binding of the proteins to all renal cell types. The target of the kidney-targeting peptide is not yet known, but ongoing proteomic studies are attempting to determine a binding partner for this peptide. More unexpected was the high binding of all proteins, including those that possess the VEGF domain, to tubular epithelial cells. This binding was not due to VEGF-receptor interactions, as these cells did not express either Flt-1 or Flk-1. Rather, this binding was possibly mediated by more generic protein reabsorption receptors, such as megalin or cubilin [57], and examining these potential interactions is also the subject of ongoing work. The in vitro binding to the tubular epithelial cells was consistent with the in vivo distribution of all of the proteins within the kidneys, which were highly concentrated in the renal tubules. Also surprising was the lack of the podocyte binding by VEGF-containing proteins, as these cells expressed high levels of VEGF receptors. However, the lack of binding to podocytes was also consistent with the in vivo distribution, as these proteins were mostly excluded from glomeruli or present only in glomerular capillaries.

The in vivo pharmacology studies revealed that the content of the proteins had strong effects on their pharmacokinetics and biodistribution. Whole-animal clearance studies showed that all additions to the core ELP molecule slowed its in vivo clearance, likely by mediating binding to target molecules. ELP and KTP–ELP predominantly accumulated in the kidneys, and KTP significantly enhanced renal deposition, which was consistent with its function as a kidney-targeting agent. ELP–VEGF, on the other hand, was present at high levels in the kidneys and in the liver. The accumulation of ELP–VEGF in the liver appeared to be species-specific, as this was shown in our previous mouse study [29] but not in rats or swine, where the kidney is the main organ of ELP–VEGF accumulation [4,23,46]. Most importantly, KTP was still effective for directing ELP–VEGF to the kidney, as kidney levels of KTP–ELP–VEGF were strongly increased relative to ELP–VEGF. We are currently evaluating the KTP–ELP–VEGF molecule in our swine model. This ongoing study will determine the therapeutic efficacy of KTP–ELP–VEGF to protect or restore the microvasculature and improve renal function in a translational model of chronic renovascular disease.

## 5. Conclusions

The ELP protein is a valuable drug carrier, and it can be modified with targeting domains and therapeutic cargo to achieve optimal drug delivery. This study demonstrated that ELP-fused cytokines retained functionality as large chimeric proteins. It also demonstrated the utility of a targeting agent to modulate the biodistribution of an ELP-fused therapeutic. Delivery of the ELP carrier and ELP-fused VEGF to the kidney was improved by the utilization of a kidney-targeting peptide. The KTP–ELP–VEGF fusion protein described here may have efficacy for treatment of multiple types of ischemic renal diseases.

## 6. Patents

The intellectual property described in this manuscript is protected by US patent number 10,322,189 and by additional pending US and worldwide patent applications.
